# Utilization of desktop 3D printer-fabricated “Cost-Effective” 3D models in orthognathic surgery

**DOI:** 10.1186/s40902-020-00269-0

**Published:** 2020-08-01

**Authors:** Masato Narita, Takashi Takaki, Takahiko Shibahara, Masashi Iwamoto, Takashi Yakushiji, Takashi Kamio

**Affiliations:** 1 Department of Oral and Maxillofacial Surgery, Tokyo Dental College, 1-2-2 Masago, Mihama-ku, Chiba, 261-8502 Japan; 2 Department of Oral Pathobiological Science and Surgery, Tokyo Dental College, 1-2-2 Masago, Mihama-ku, Chiba, 261-8502 Japan; 3 Oral and Maxillofacial Surgery, National Hospital Organization Takasaki General Medical Center, 32 Takamatsu, Takasaki, Gunma 371-0829 Japan; 4 Department of Oral and Maxillofacial Radiology, The Nippon Dental University, 1-9-20 Fujimi-cho, Chiyoda-ku, Tokyo, 102-8159 Japan

**Keywords:** 3D printer, 3D CAD, Patient specific, Orthognathic surgery, Jaw deformity

## Abstract

**Background:**

In daily practice, three-dimensional patient-specific jawbone models (3D models) are a useful tool in surgical planning and simulation, resident training, patient education, and communication between the physicians in charge. The progressive improvements of the hardware and software have made it easy to obtain 3D models. Recently, in the field of oral and maxillofacial surgery, there are many reports on the benefits of 3D models. We introduced a desktop 3D printer in our department, and after a prolonged struggle, we successfully constructed an environment for the “in-house” fabrication of the previously outsourced 3D models that were initially outsourced. Through various efforts, it is now possible to supply inexpensive 3D models stably, and thus ensure safety and precision in surgeries. We report the cases in which inexpensive 3D models were used for orthodontic surgical simulation and discuss the surgical outcomes.

**Review:**

We explained the specific CT scanning considerations for 3D printing, 3D printing failures, and how to deal with them. We also used 3D models fabricated in our system to determine the contribution to the surgery. Based on the surgical outcomes of the two operators, we compared the operating time and the amount of bleeding for 25 patients who underwent surgery using a 3D model in preoperative simulations and 20 patients without using a 3D model. There was a statistically significant difference in the operating time between the two groups.

**Conclusions:**

In this article, we present, with surgical examples, our in-house practice of 3D simulation at low costs, the reality of 3D model fabrication, problems to be resolved, and some future prospects.

## Background

The success of orthognathic surgery depends on appropriate physical examination, accurate diagnosis, and treatment planning. When developing a treatment plan, it is necessary to diagnose the dentofacial deformities accurately using conventional facial photographs, cephalometric analysis such as two-dimensional (2D) modalities, and dental casts mounted on an articulator with face-bow transfer [1]. In order to reproduce the treatment plan during surgery, a mock trial is performed using dental casts to simulate the occlusion and jaw movements. Remarkable advances made in recent times in the field of three-dimensional (3D) engineering have benefitted the development of 3D virtual surgery planning on a personal computer (PC). In recent years, 3D simulations using multidetector-row computed tomography (MDCT) and/or limited cone-beam computed tomography (CBCT) have been performed in many facilities for orthognathic surgery [2]. Around the same time, 3D printing technology became familiar, and it became easy to perform simulated surgery using 3D models that reproduced the patient-specific morphology of the jaws. 3D models reveal the complicated configuration of the teeth and jaws and can guide surgical simulations such as osteotomy, plate vending, and screw fixation [3, 4].

Until recently, obtaining 3D models was expensive and thus used mainly for patients with severe deformities. At the end of 2014, we launched a “one-stop 3D printing lab,” an environment where data creation and 3D model fabrication can be performed in one facility [5]. This “one-stop 3D printing lab” has made it possible to obtain 3D models immediately and inexpensively. Today, the fabricated 3D models are used not only for surgical simulation but also for creating a shared understanding among the surgical team, patient education, medical/dental education, and so on.

In this article, we present an overview of “low cost” 3D model production, our efforts to fabricate 3D models, and discuss the future of 3D models in orthognathic surgery.

## Review

### 3D printing workflow

**Fig. 1:**
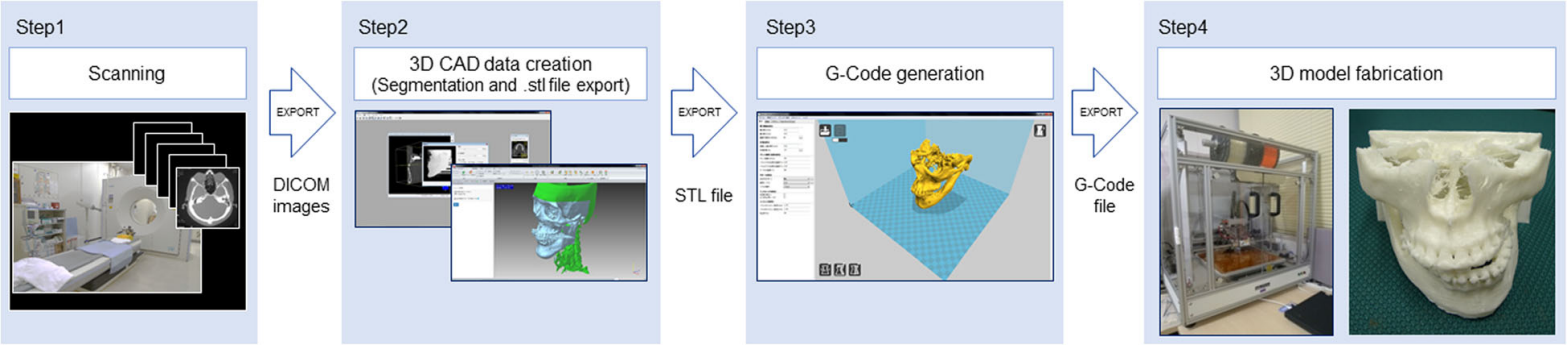
Process workflow for 3D model fabrication using desktop 3D printers. (Step 1) MDCT scanning. (Step 2) Open in medical image processing software “Volume Extractor 3.0” and polygon data editing software “POLYGONALmeister” to check, create, and adjust the 3D CAD model. (Step 3) Open in 3D printer slicing software “CURA” to prepare and generate supports. (Step 4) Fabrication with MF-2000

Figure 1 shows our 3D fabrication workflow. After MDCT scanning, we segment and create STL data from the DICOM images, generate G-Code for 3D printable data, and then perform 3D fabrication.

#### MDCT scanner and scanning parameters

**Fig. 2:**
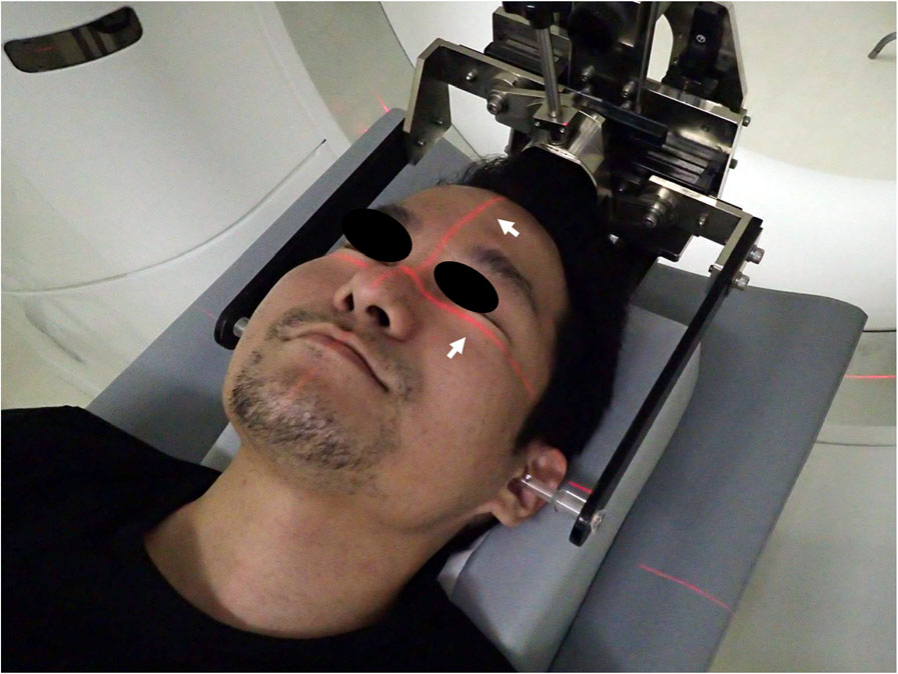
Bilateral ear-rods made according to the cephalogram. The head position is defined using the ear rod and guide beam (arrow). Scanning with ear-rods helps to define the position of the head (parallelism) and makes STL data creation easier

In addition to the fabrication of 3D models for surgical simulation, MDCT scanning assists in evaluating the jawbone morphology. MDCT is performed approximately 1 month before the surgery. The orthodontic archwire is removed before the MDCT scanning procedure. Bilateral ear-rods, similar to that used in cephalograms, are positioned to stabilize the head during scanning, thus limiting horizontal differentiation (Fig. 2). This makes it easier to define the occlusal plane, i.e., the spread of metal artifacts due to the intraoral metal (dental prosthetics and orthodontic devices, etc.) is focused as narrowly as possible. This helps to reduce the amount of effort (manual removal of noise on the screen) during segmentation and STL data creation for DICOM image data. MDCT scanning is performed at the final phase of swallowing in a resting occlusal position. In skeletal class II cases, the jaw position may vary from that at the time of cephalometric radiography. Therefore, if necessary, the jaw position may be determined using a pre-prepared resin block. MDCT scanning was performed with a 64-slice MDCT scanner (Somatom Definition AS64, Siemens, Erlangen, Germany) with the following parameters: 120-kV tube voltage, 110 mAs, 0.6-mm slice thickness, and 250-mm field of view (FOV).

#### DICOM segmentation and STL creation

The acquired MDCT images, i.e., DICOM images, are converted into 3D CAD (computer-aided design) data by medical image processing software. The region of interest (ROI) of 3D CAD data is segmented, and image binarization is performed for processing the CT slice images. Using a 3D image processing software package (Volume Extractor 3.0, i-Plants systems, Iwate, Japan) [6], a continuous slice image is segmented into an STL file format that enables three-dimensional fabrication, i.e., polygon data. Next, the polygon data editing software (POLYGONALmeister Ver.4; UEL Corp., Tokyo, Japan) is used for the ROI setting and reducing the data volume without changing the shape.

#### 3D printing system

**Fig. 3:**
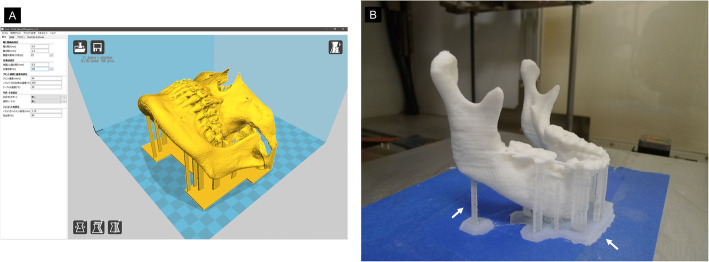
3D CAD model as displayed in 3D printer slicing software “CURA.” Arrows indicate support structures for increasing the fabricating stability. The ease of removal of the support structures changes depending on the printing parameter setting and the installation position

In our system, STL data cannot be sent directly to a 3D printer. Therefore, it is necessary to export the STL data into G-Code data that is widely used in numerical control programing language [7, 8]. This was achieved using the open-source G-code generator slicing software package (CURA Ver.15.04, Ultimaker, Geldermalsen, Netherlands). This software provides parameters suitable for 3D printers, such as generating support structures for 3D models, setting the temperature during printing, determining the printing direction, and setting the printing speed and infill density of the filament (Fig. 3).

**Fig. 4:**
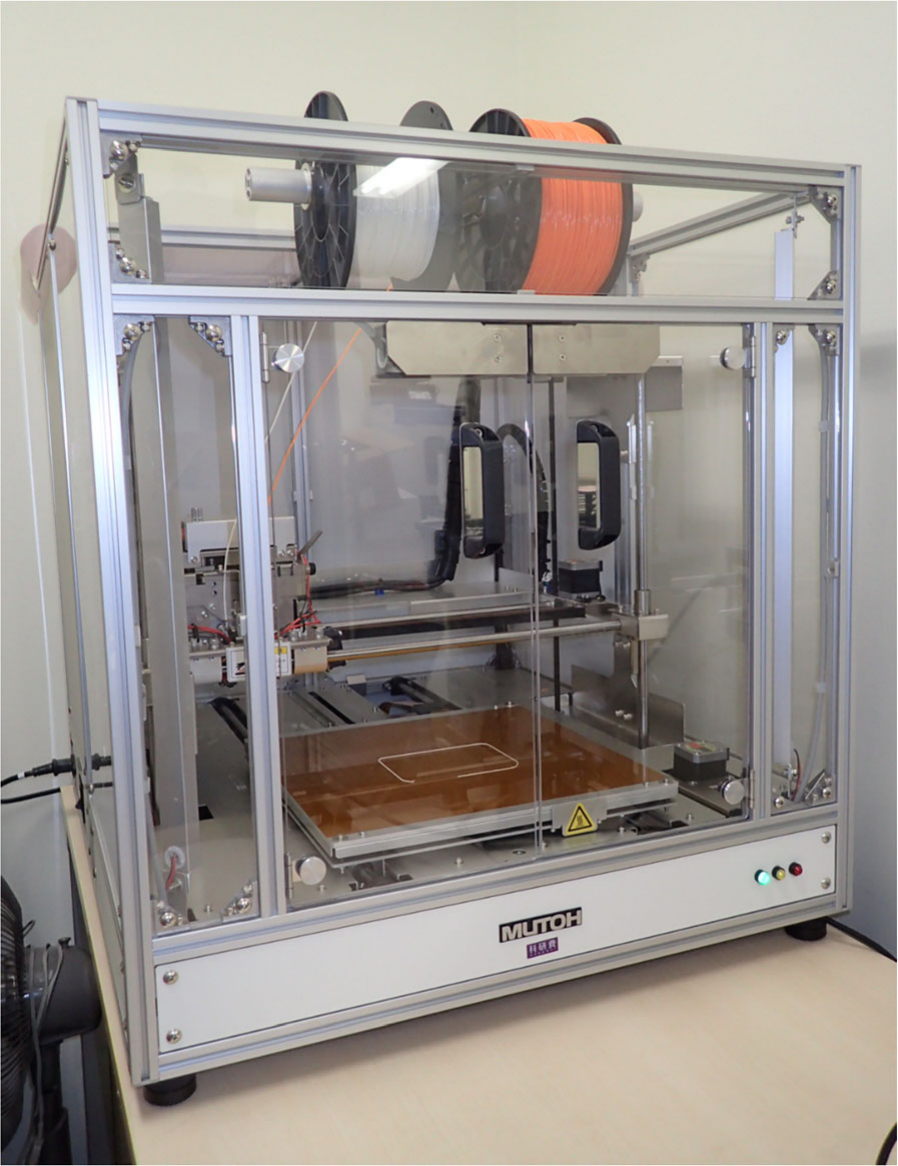
Desktop FDM 3D printer “Value3D MagiX MF-2000”

In jaw deformity cases, we use the desktop-fused deposition modeling (FDM) 3D printer (Value3D MagiX MF-2000, MUTOH Industries Ltd., Tokyo, Japan) with a maximum printing size of 30 cm × 30 cm × 30 cm (Fig. 4). PLA (polylactic acid) filament, a vegetable-based plastic material with considerable strength and dimensional stability, is used [9]. The 3D printing parameters were as follows: Filament used: Pxmalion, 1.75-mm PLA, lamination pitch: 0.3 mm, infill density: 20%, printing speed: 30-50 mm/s, with support structures and a raft.

### 3D printing failures and solutions

**Fig. 5:**
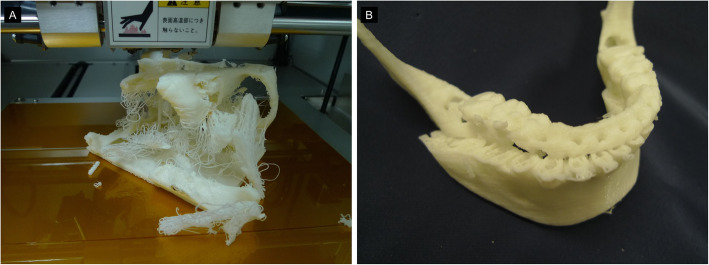
Fabrication failure. **a** A failed 3D model that came off the bed (heat table) in the middle of fabricate and continued to laminate, resulting in a failed 3D model. **b** This is due to the detached from the bed (heat table) during the fabricate

We have fabricated 3D models of 93 cases with jaw deformities, and have experienced many diverse 3D printing failures (Fig. 5). We looked for resources on the Internet to resolve similar challenges faced by people around the world. Table 1 shows the problems encountered and their remedies.

**Table 1:** Common problems encountered in 3D printing

**A. Possible causes and remedies of 3D printing failure due to inadequate 3D CAD data creation**		
	1.	Ingenuity during CT scanning—ingenuity during imaging to minimize metal artifacts
	2.	Design changes suitable for FDM 3D printer—3D CAD data creation that understands the characteristics of FDM 3D printer
	3.	Utilization of STL editing software—noise reduction on image, data volume reduction, loss compensation, etc.
	4.	Add the support structures—building in 3D CAD data creation
**B. Possible causes and remedies of 3D printing failure due to 3D printing software (parameters of the slicing software)**		
	1.	Adjustment of print parameters suitable for each 3D printer
	2.	Adjustment of print temperature according to each filament
	3.	Adjustment of support structure settings for 3D printing
	4.	Using other 3D printing software
**C. Possible causes and remedies of 3D printing failure due to 3D printer**		
	1.	Extruder (the part of the 3D printer that ejects material in semi-liquid) adjustment and/or replacement
	2.	Using and/or replacing other filaments
	3.	Using and/or replacing adhesive sheet/materials of heat beds that makes the cooling 3D models
	4.	Responding to temperature—room temperature adjustment and ventilation from the surroundings during 3D printing

### Practical use of 3D models for orthognathic surgery “Is it cost effective?”

**Fig. 6:**
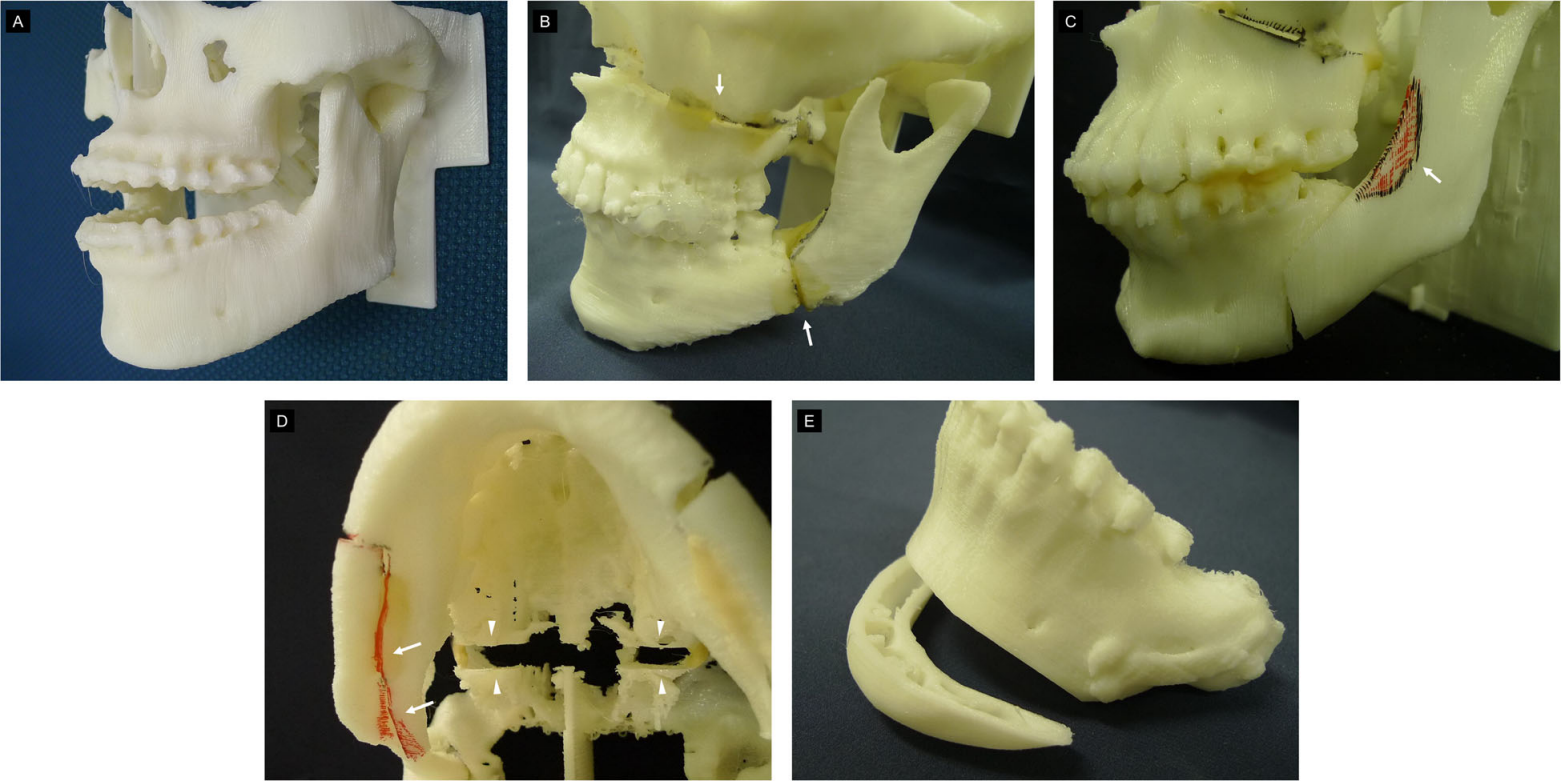
3D models used for orthognathic surgery, fabricated with a desktop 3D printer and surgical simulation. Since the FOV is from the orbital floor to the lower edge of the mandible, the slice thickness is greater. Therefore, the reproducibility of the morphology of the teeth is poor, but the accuracy is sufficiently high for osteotomy simulation. **a** 3D model with maxillary retrusion and mandibular protrusion. **b** Surgical simulation of Le Fort I osteotomy and SSRO (arrow). **c** By performing 3D model surgery, the amount of trimming of the anterior mandibular ramus can be predicted (arrowheads). **d** The arrowhead shows the amount of maxilla movement. The arrow shows bone interference. **e** In genioplasty, checking the width and height of bones and the position of mental foramen with a 3D model is very useful for predicting risk

Figure 6a-e shows the surgical simulation of Le Fort I osteotomy and sagittal split ramus osteotomy (SSRO) using 3D models. 3D models engage the sight and touch of the doctors. They provide them with an understanding of the anatomical structures in advance, thus offsetting the issues caused by limited visualization during surgery [5]. Unlike plaster models, 3D models provide information about certain anatomical structures like the nasal floor and mandibular ramus.

Machine tools are not used to cut bones, and thus, the 3D model must be efficient enough to be cut through with surgical equipment/instruments. If the 3D model is solid, cutting through would be difficult, and if it is fragile, it will break. The PLA 3D models that we fabricated are similar to the dental resin; therefore, the 3D models have good affinity and operability with dental equipment/instruments. Sterilization of these 3D models does not deteriorate or deform their structure. Surgical splints can be created using CAD/CAM [10, 11]; however, due to time constraints and a high number of surgeries, we manually prepared the surgical splints. Obviously, the greater the number of operations, the less time is spent on model operations using 3D models.

Based on the surgical outcomes of the two operators who completed Le Fort I osteotomy and SSRO, we compared the operating time and the amount of bleeding for 25 patients who underwent surgery using a 3D model in preoperative simulations and 20 patients without using a 3D model. Lack of a 3D model simulation implies a time when there were no 3D printers or they were not easily available. Although it is possible that the operators’ surgical skills could have improved over time, there was a statistically significant difference in the operating time between the two groups (Table 2). We are aware that it is extreme to discuss surgical outcomes based solely on “with or without a preoperative 3D model simulation.” As per our experience, the time spent on trial and error during surgery can be reduced if the equipment is prepared before surgery (e.g., preparing plates and screws to be used and bending plates). A safer operation can reduce the amount of bleeding, which in turn contributes to a reduction in operating time.

**Table 2:** Comparison of surgical outcomes with and without 3D models. Welch’s *t* test was used to test the difference between the mean values of amount of bleeding and the operating time

	With 3D models	Without 3D models	*p* value
Number of cases	25	20	
Mean amount of bleeding	252.2 ± 97.7 g	331.2 ± 85.9 g	0.0971
Mean operating time	226 ± 18 min	260 ± 36 min	0.0255

### Obtaining 3D models and considering its cost

An overview of the 3D models that we fabricated for the preoperative simulation of orthognathic surgery is shown in Table 3. The time required for fabrication depends on the size of the 3D model and the printing parameters; therefore, the cost of the 3D model varies greatly depending on these factors. It is also affected by the price and type of filament used. The average weight (without support structures) of our 3D model was approximately 165 g. Calculating this from the cost of the filament, the price of one 3D model is approximately 5 US dollars. This does not include the labor and delivery costs involved in 3D CAD data creation. Additionally, obtaining an immediate 3D model of satisfactory quality increased both, the fabrication time and the failure rate. Nowadays, the number of medical image modeling service companies is increasing, and ordering from such companies has become easier. Considering the time required for data creation and fabrication, such a service may be useful.

**Table 3:** Overview of the fabrication of our 3D models in orthognathic surgery

Number of cases	Mean time required for fabrication	Mean weight of the fabricated 3D model	Mean cost per 3D model
92	12 h 14 m	166.5 g	5.2 USD

As there are few reports of using FDM 3D printers for orthognathic surgery, much less similar studies and reports on fabrication costs [12–14], although comparisons at the same level are difficult, we believe that our system is inexpensive and more economical than these reports.

### Need of addressing unsolved problems of 3D models

When discussing 3D models, its accuracy and economics must also be mentioned. As shown in a previous study using the same 3D printers and filaments [5], the accuracy of the 3D models depends on various factors, including the spatial resolution of the MDCT, the quality of the STL data, and the setting of the 3D printer, such as the lamination pitch, printing temperature, and the performance of the filament used. The minimum lamination pitch of the 3D printer used by our facility was 0.1 mm. Our 3D printers can print as designed. However, as the voxel size of the MDCT scan is 0.49 × 0.49 × 0.6 mm, it is difficult to acquire precision of the occlusal surface of the teeth due to the limitation of spatial resolution. Therefore, occlusion has been conventionally determined using a plaster model. It should be emphasized that our 3D models do not replace these plaster models, but are used in conjunction. Metal artifacts caused by dental prostheses and/or orthodontic appliances also affect the scan images. From the perspective of X-ray exposure, frequent CT scans should be avoided for 3D model fabrication. This is possible with the use of an intraoral scanner, which does not involve X-ray exposure and can obtain detailed surface information of oral structures such as teeth [15, 16]. If it became easier to superimpose MDCT and/or CBCT data with data from an intraoral scanner, the lack of morphological information due to metal artifacts can be compensated. However, at present, it is not easy to superimpose data of the two modalities, and the intraoral scanner is still expensive.

It is important to shorten the time required for in-house fabrication. The ability to fabricate 3D models at low cost will, in turn, increase the quantitative production. This makes it easier to perform multiple surgical procedures, such as the difference between the osteotomy lines in SSRO. Thus, the use of desktop 3D printers is likely to increase. Consecutively, this demands constant updates about the significant 3D engineering development. In addition, it is necessary to deepen the familiarity with software operation, 3D printers, and printing materials. For this reason, we believe it is meaningful to study 3D engineering at the pre-graduate level of medical/dental education.

## Conclusions

In this article, we present, with surgical examples, our in-house practice of 3D simulation at low costs, the reality of 3D model fabrication, problems to be resolved, and some future prospects. With the development of 3D printing technology, we can now obtain the previously expensive patient-specific 3D models at a low cost. 3D printing technology can be applied not only to orthognathic surgery but also to other aspects of oral and maxillofacial surgery. We hope our attempt initiates further related discussions.

## Data Availability

The datasets used during the current study are available from the corresponding author on reasonable request.

## Abbreviations

2D: Two-dimensional
3D: Three-dimensional
CAD: Computer-aided design
CAD/CAM: Computer-aided design/computer-aided manufacturing
CBCT: Cone-beam computed tomography
CT: Computed tomography
DICOM: Digital imaging and communications in medicine (file format)
FDM: Fused deposition modeling
FOV: Field of view
MDCT: Multidetector-row-computed tomography
PLA: Polylactic acid
ROI: Region of interest
SSRO: Sagittal split ramus osteotomy
STL: Stereolithography (file format)
